# Major Bleeding Rates Associated With Catheter-Directed Therapies in Patients With Intermediate-Risk Pulmonary Embolism

**DOI:** 10.1016/j.jacadv.2026.102623

**Published:** 2026-03-25

**Authors:** Christian Bichard, Ka U. Lio, Vladimir Lakhter, Parth Rali, Huaqing Zhao, Riyaz Bashir

**Affiliations:** a Department of Internal Medicine, Temple University Hospital, Philadelphia, Pennsylvania, USA; b Department of Pulmonary, Allergy, Critical Care, and Sleep Medicine, Emory University Hospital, Atlanta, Georgia, USA; c Department of Cardiovascular Diseases, Temple University Hospital, Philadelphia, Pennsylvania, USA; d Department of Thoracic Medicine and Surgery, Temple University Hospital, Philadelphia, Pennsylvania, USA; e Department of Clinical Sciences, Temple University Hospital, Philadelphia, Pennsylvania, USA; f Department of Cardiovascular Medicine, Mayo Clinic, Jacksonville, Florida, USA

**Keywords:** catheter-directed lysis, intermediate-risk pulmonary embolism, mechanical thrombectomy, pharmacomechanical lysis

**What is the clinical question being addressed?**

What are the comparative major bleeding rates with various catheter-based therapies for intermediate-risk pulmonary embolism?

**What is the main finding?**

Pharmacomechanical lysis with the Bashir Endovascular Catheter had the lowest major bleeding rates compared with ultrasound-assisted thrombolysis and mechanical thrombectomy.

Acute pulmonary embolism (PE) is the third leading cause of cardiovascular death, with an incidence of 39 to 115 per 100,000 annually and up to 100,000 deaths per year in the United States. Intermediate-risk PE, defined by right ventricular dysfunction and/or biomarker elevation without shock, is associated with a short-term mortality of 10% despite anticoagulation. Catheter-based therapies such as ultrasound-assisted thrombolysis (US-CDT), large-bore mechanical thrombectomy (LBMT), and pharmacomechanical lysis (PML) using the Bashir Endovascular Catheter (BEC) are increasingly used in contemporary clinical practice.

The BEC is a newer device with a distinct mechanical and pharmacological mechanism of action. The mechanical expansion of the nitinol scaffolding fissures the thrombus and embeds the infusion limbs deep within the clot. Then, a high-pressure pulse spray of dilute, low-dose recombinant tissue plasminogen activator (rtPA) is administered within the clot, thereby prolonging the half-life of fibrin bound rtPA. This approach is particularly effective in treating distal segmental artery occlusions while reducing the systemic exposure of rtPA.^1^, ^2^, ^3^ This study aimed to evaluate real-world comparative major bleeding rates, as defined by the International Society on Thrombosis and Haemostasis (ISTH) criteria, among the most frequently used catheter-based treatments in intermediate-risk PE patients at our institution.

We retrospectively analyzed the Temple University Hospital Pulmonary Embolism Response Team (PERT) registry (2017-2022), which includes adults with intermediate-risk PE treated with US-CDT, PML, or LBMT. The primary endpoint was major bleeding according to the ISTH definition; secondary endpoints included Global Use of Strategies to Open Occluded Coronary Arteries and Valve Academic Research Consortium-2 major bleeding rates, rates of intracranial hemorrhage, all-cause mortality at 30 days, and blood transfusion rates. Our study received proper ethical oversight and institutional review board approval was obtained. To evaluate the effectiveness of BEC, CDT, and LBMT, propensity score–based inverse probability of treatment weighting (IPTW) was applied to balance baseline and follow-up characteristics and minimize selection bias. Age, body mass index, diabetes, simplified PE severity index, HAS-BLED (hypertension, abnormal renal or liver function, stroke history, bleeding history or predisposition, labile international normalized ratio, elderly, drugs or alcohol use), registro informatizado de enfermedad tromboembolica score, troponin, brain natriuretic peptide, and right ventricular strain were included. Propensity scores were estimated using gradient-boosted models, which flexibly capture nonlinear relationships and interactions among covariates and are well suited for balancing multiple treatment groups and enabling pairwise comparisons. IPTW were constructed based on the estimated propensity scores using the twang package in R, with stabilized weights incorporating the marginal probability of treatment assignment to improve weight stability. The weighted analyses were conducted using the survey package, and covariate balance after weighting was assessed using standardized mean differences. Logistic regression was implemented for all binary outcome analyses. Specifically, before weighting, unadjusted associations between treatment groups and outcomes were estimated using logistic regression in R. After propensity score–based IPTW using the mnps() function from the twang package, weighted logistic regression models were fitted using svyglm to estimate treatment effects. The primary reported estimates were obtained from IPTW-weighted logistic regression without additional covariate adjustment. Sensitivity analyses additionally included age as a covariate in the weighted logistic regression models to assess robustness of the results.

Among a total of 461 intermediate-risk PE patients for which the PERT was activated, 104 (23%) underwent catheter-based therapy: 49 (47%) US-CDT, 24 (23%) PML, and 31 (30%) LBMT. After IPTW adjustment, major bleeding by ISTH criteria occurred in 19.3 of 57 patients (34%) of LBMT, 14 of 83 (17%) of US-CDT, and 0 of 63 (0%) of PML. Compared with PML, odds of major bleeding were higher for US-CDT (OR: 10.3; 95% CI: 1.1-93.6; *P* < 0.05) and LBMT (OR: 25.6; 95% CI: 2.8-231.1; *P* < 0.05). Major bleeding rates by Global Use of Strategies to Open Occluded Coronary Arteries and Valve Academic Research Consortium-2criteria showed a similar trend (Figure 1). One intracranial hemorrhage occurred in the LBMT group; none occurred in the other groups. Thirty-day mortality was 4% (2/49) in US-CDT, 4% (1/24) in PML, and 6% (2/31) in LBMT groups, with 1 procedure-related death in the LBMT group. Blood transfusion rates were also higher in the LBMT group without reaching statistical significance.

**Figure 1:**
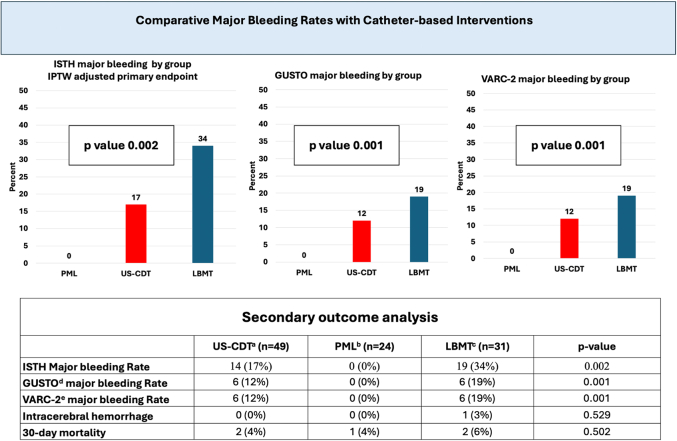
**Comparison of Major Bleeding Rates According to ISTH, GUSTO, and VARC-2 Criteria for US-CDT, PML, and LBMT, After IPTW Adjustment** All P values are IPTW adjusted. GUSTO = Global Strategies for Opening Occluded Coronary Arteries; ISTH = International Society on Thrombosis and Haemostasis; LBMT = large-bore mechanical thrombectomy; PML = pharmacomechanical lysis; US-CDT = ultrasound-assisted catheter-directed thrombolysis; VARC-2 = Valve Academic Research Consortium-2.

The reduced major bleeding in the PML group may be related to its mechanical mechanism of action, which allows for the use of a low dose of rtPA delivered within the thrombus [1,2]. The higher rates of major bleeding observed in our study compared to those reported in the PEERLESS (Pulmonary Embolism Extraction with the FlowTriever System versus Catheter-Directed Thrombolysis) trial (∼6.9%)^4^ may be attributed to differences in the trial design and operator experience. Patients in randomized trials are prospectively enrolled and treated by experienced operators, whereas real-world observational cohorts consistently demonstrate higher bleeding with LBMT,^5^ possibly reflecting learning curve. Our results, therefore, align more closely with large observational analyses, which have consistently suggested higher bleeding rates in real-world clinical practice.

Limitations include small sample size, a single-center setting, and potential for unmeasured confounding. Treatment allocation was determined by the on-call PERT physicians, introducing possible indication bias based on device availability, physician preference, or clinical profile. Another limitation is that IPTW balanced covariates and the differences in baseline bleeding risks may not have been fully accounted for. This highlights the need for larger multicenter data sets and randomized trials to establish comparative safety more definitively.

In conclusion, PML with BEC was associated with low major bleeding, whereas US-CDT and LBMT carried a significantly higher risk of major bleeding. These results suggest that PML may be a safe catheter-based therapy for patients with intermediate-risk PE, who are at high risk of bleeding, although confirmation in larger randomized studies is needed.

## Funding support and author disclosures

Dr Lakhter has received consulting fees/honorarium from 10.13039/501100008645Terumo, Thrombolex, Neptune Medical, and Magneto. Dr Bashir has equity interest in Thrombolex Inc. and received 10.13039/100000050NHLBI funding. All other authors have reported that they have no relationships relevant to the contents of this paper to disclose.
